# Parisian Medical Schools

**Published:** 1850-05

**Authors:** 


### EDITORIAL DEPARTMENT.

*Parisian Medical Schools.*—The accomplished editor of the Ohio Medical and Surgical Journal, in an editorial article on the subject of free schools, offers some remarks on the opportunities for acquiring medical knowledge at Paris, which are deserving special attention, inasmuch as he speaks from ample personal observation. It is one of the ridiculous notions of the day, that a sojourn at Paris is indispensible to the student who desires to perfect himself in the attainments constituting a sound medical education. Nothing can be plainer than the absurdity of such an opinion. The subjects of medical knowledge are less dependent than those of any other pursuit, on circumstances peculiar to any country. The laws of disease, and the principles of surgery, invite investigation wherever there are human beings, since in every place and age they are exposed to accidents, and the various ills that flesh is heir to. The materials for study, and the conditions for proficiency, are abundantly available without need of crossing the Atlantic. And we may rest assured that with the present facilities for the publication of newly developed observations, and the latest conceived hypotheses, it is quite unnecessary to go to Paris to learn what our distinguished brethren there are doing, or of what they are thinking. Their anxiety that their most remote admirers should be promptly informed on these points, is probably not less than the avidity of the latter to obtain this information. The large hospitals of the French metropolis doubtless furnish a fine field for obtaining practical acquaintance with pathology and diagnosis. We have no wish to disparage their advantages, but there is no lack of similar opportunities in this country, and there, as here, the richness of the field alone will not ensure an abundant harvest, so much as the industry and skill of the husbandman. In the latter lies the secret of improvement and eminence. It is of little avail, merely to have been where application might have accomplished the most; and well directed, persevering efforts, will more than make amends, anywhere, for paucity of materials for improvement. We certainly would not discourage a sojourn at Paris by those who have time and money to spare; but we do hold it to be more than ridiculous to attach any importance to the mere fact of having studied the phenomena of disease in Europe instead of America! It diminishes the respect of medical students and the medical Profession for the benefits to be enjoyed in our own country, to invest those of foreign lands with a fictitious or fashionable pre-eminence. Its effect upon those who resort to foreign countries is bad, since by leading them to over-estimate the value of mere *place* in the prosecution of their studies, they naturally under-value the importance of their own exertions.

If it be indeed true, (as some appear to think,) that there are insuperable difficulties in the way of acquiring medical knowledge, and advancing scientific investigations in this country, the fault is not with what surrounds us, but with ourselves—it is subjective, not objective; and the inference follows, either that medicine is an exotic which cannot thrive on American soil, or that in this, if not in other branches of knowledge, the American mind evinces inferiority by comparison with the Gallic. We doubt if our readers will be satisfied to rest on either of the horns of this dilemma; and, if not, they must concur in the opinion, that if medical education and knowledge with us is not what it is elsewhere, we have no valid excuse therefor, since there is no reason why we should not emulate, if not excel other nations in this, as in other achievements dependent mainly on human exertion, stimulated by motives which should be not less active with us than abroad. If medicine in this Republic falls below the French standard, it does not hold the position to which it may and should aspire; and a patriotic regard for our national character and institutions should prevent us from being satisfied with our condition, until an equality, at least, is secured.

We append the portion of the article by Prof. Smith, which has suggested the foregoing remarks:—

While on the subject of French schools, we will take the opportunity to express our opinion of the true position of Paris as a seat of medical learning, and that is—that no man who has not acquired a sound and thoroughly practical knowledge of medicine, and who is not in the habit of thinking for himself, should go to Paris to improve his knowledge. We have known many a promising physician utterly blighted and spoiled thereby. Paris is the place to study specialities in; to *study for oneself*, not to learn from others merely, for the Parisian "celebrities," teach not only all that is known, but considerable that is *not* known, and the practice of the different physicians in the same hospital, is commonly most contradictory. The hospitals for special diseases are, however, by no means so easy of access as is supposed; but a limited number of students being permitted to enter them, and that, in some cases, under great restrictions. The very crowd of students is a great disadvantage, particularly to a foreigner not speaking French like a native ; for every foot of distance intervening between him and the speaker, renders it more difficult to follow the discourse. The professional morality of many great surgeons does not come up to our standard. We have seen most barberous and unjustifiable operations in Parisian hospitals ; and we have heard Lisfranc devote the greater part of what should be a clinical lecture, to abuse of Velpeau, who in his turn would pay back Lisfranc in the same coin, to the great amusement if not improvement of the audience.

By making friends with the internes end externes, (answering to the house-surgeons and dressers of the English hospitals,) pathological anatomy may be studied with considerable advantage, from the great number of post-mortem examinations that are made. There are also fine opportunities for the investigation of disease of the skin, and some of those of women, and the much neglected branch of Orthopedy. The art of diagnosis has been brought to a most wonderful degree of precision by the French ; indeed, we used sometimes to think, judging from the treatment ordered, that the study of the natural history of disease, not the best mode of curing it, was the object in view, both of professors and pupils, who seemed almost to wish the death of the patient, that they might have an opportunity of verifying the diagnosis. Practical anatomy may be studied to one's heart's content, the supply of material being abundant and cheap, but of the arrangement of the dissecting rooms with regard to cleanliness and comfort, unless much changed of late, "least said soonest mended." Regular dissections are not allowed during the summer months, but the subjects brought in are given to the young men to practice surgical operations upon. In one thing Paris is pre-eminent just now, and that is, in facilities for learning the use of the microscope, and studying microscopic anatomy. Gruby and others give regular demonstrative private courses on this subject, which are most instructive and agreeable ; indeed, the private courses of the internes in the hospitals, and of many distinguished men out of them, for which a smaller fee is charged, are generally speaking, far more profitable to the student than the public *free* lectures of the salaried Professors.

